# Contrast-to-noise ratios and thickness-normalized, ventilation-dependent signal levels in dark-field and conventional *in vivo* thorax radiographs of two pigs

**DOI:** 10.1371/journal.pone.0217858

**Published:** 2019-06-03

**Authors:** Fabio De Marco, Konstantin Willer, Lukas B. Gromann, Jana Andrejewski, Katharina Hellbach, Andrea Bähr, Michaela Dmochewitz, Thomas Koehler, Hanns-Ingo Maack, Franz Pfeiffer, Julia Herzen

**Affiliations:** 1 Chair of Biomedical Physics & School of BioMedical Engineering, Technical University of Munich, Garching, Germany; 2 Department of Radiology, University Hospital, LMU Munich, Munich, Germany; 3 Institute of Molecular Animal Breeding and Biotechnology, LMU Munich, Oberschleissheim, Germany; 4 Philips GmbH Innovative Technologies, Research Laboratories, Hamburg, Germany; 5 Institute for Advanced Study, Technical University of Munich, Garching, Germany; 6 Philips Medical Systems DMC GmbH, Hamburg, Germany; 7 Department of Diagnostic and Interventional Radiology, School of Medicine & Klinikum rechts der Isar, Technical University of Munich, Munich, Germany; Lunds Universitet, SWEDEN

## Abstract

Lung tissue causes significant small-angle X-ray scattering, which can be visualized with grating-based X-ray dark-field imaging. Structural lung diseases alter alveolar microstructure, which often causes a dark-field signal decrease. The imaging method provides benefits for diagnosis of such diseases in small-animal models, and was successfully used on porcine and human lungs in a fringe-scanning setup. Micro- and macroscopic changes occur in the lung during breathing, but their individual effects on the dark-field signal are unknown. However, this information is important for quantitative medical evaluation of dark-field thorax radiographs. To estimate the effect of these changes on the dark-field signal during a clinical examination, we acquired *in vivo* dark-field chest radiographs of two pigs at three ventilation pressures. Pigs were used due to the high degree of similarity between porcine and human lungs. To analyze lung expansion separately, we acquired CT scans of both pigs at comparable posture and ventilation pressures. Segmentation, masking, and forward-projection of the CT datasets yielded maps of lung thickness and logarithmic lung attenuation signal in registration with the dark-field radiographs. Upon correlating this data, we discovered approximately linear relationships between the logarithmic dark-field signal and both projected quantities for all scans. Increasing ventilation pressure strongly decreased dark-field extinction coefficients, whereas the ratio of lung dark-field and attenuation signal changed only slightly. Furthermore, we investigated ratios of dark-field and attenuation noise levels at realistic signal levels via calculations and phantom measurements. Dark-field contrast-to-noise ratio (CNR) per lung height was 5 to 10% of the same quantity in attenuation. We conclude that better CNR performance in the dark-field modality is typically due to greater anatomical noise in the conventional radiograph. Given the high physiological similarity of human and porcine lungs, the presented thickness-normalized, ventilation-dependent values allow estimation of dark-field activity of human lungs of variable size and inspiration, which facilitates the design of suitable clinical imaging setups.

## Introduction

Grating-based X-ray phase-contrast and dark-field imaging typically exploits the occurrence of periodic intensity patterns at certain positions downstream of a grating, which is introduced in the beam before or after a sample. In addition to the conventional attenuation image, analyzing the distortion of these intensity patterns allows retrieval of information about X-ray refraction and small-angle scattering by the sample [1–5]. In particular, the three-grating Talbot-Lau interferometer enables the use of lab-based high-flux, low-coherence X-ray sources [6].

Small-angle scattering information is encoded in the so-called dark-field modality, which is highly sensitive to microstructural fluctuations [4]. Dark-field signal strength depends on the autocorrelation function of the sample’s electron density distribution. For mono-energetic radiation, the projection of this function in beam direction, evaluated at a value given by grating periods, X-ray wavelength and sample position in the beam, determines dark-field signal magnitude [7, 8].

Grating-based phase-contrast/dark-field imaging and closely related coded-aperture techniques [9, 10] have significant potential for application in many disciplines, such as non-destructive testing [11, 12], as well as biomedical and preclinical imaging, such as mammography [13–16] and pulmonary imaging [17–23]. Concerning the latter, X-ray dark-field radiography was found to provide benefits for the diagnosis of emphysema [18–20], fibrosis [21], neonatal lung injury [22], and lung cancer [23] in small-animal models.

It is therefore of great interest to determine whether these benefits can be replicated in large-animal models and preclinical trials. However, the necessary adaptation of grating-based imaging setups is inhibited by several technical challenges: The field of view (FOV) achievable with the method is usually confined by the limited size of the required gratings, and acquisition times typically far exceed those of conventional radiography. These limitations can however be ameliorated by tiling of multiple gratings into larger structures [24], as well as the application of scanning acquisition techniques [25]. Using these approaches, significant progress was recently made in the development of setups for the acquisition of large-FOV dark-field and phase-contrast radiographs with acquisition times of the order of one minute, both with a primary focus on non-destructive testing and biomedical imaging [26–30]. Furthermore, the possibility to implement dark-field imaging functionality to existing medical imaging hardware, such as a scanning mammography system [26] and a C-arm setup [31], has been demonstrated.

The setup in [27] was used for acquisition of the first *in vivo* X-ray dark-field images of a porcine thorax, demonstrating the successful translation of dark-field imaging to a large-animal model. With the same device, it was shown that the dark-field modality achieves systematically higher contrast-to-noise (CNR) values than comparable attenuation radiographs for lateral pneumothoraxes induced on pigs both *in vivo* and *ex vivo* [32].

Furthermore, the setup was used for a study on a deceased human body, which demonstrated the compatibility of the imaging technique with medical application [33]. For pigs (and likely humans), signal strength varies considerably between different stages of the breathing cycle. Since the medical promise of pulmonary dark-field radiography lies in detecting pathological microstructural changes, it is of major importance to distinguish these from normal changes induced by breathing.

In dark-field radiography, signal strength is described by a line integral through the entire lung, along the direction of projection. The contribution of a given volume element to this integral however depends on local microstructure, specifically on the autocorrelation function values of electron density at a few micrometers or less [7, 8]. Dark-field signal strength is thus a function of structural parameters on multiple length scales.

Lungs consist of hierarchical airway and blood vessel networks, with relevant length scales ranging from centimeters for the bronchi, down to a few micrometers for alveolar walls (septum). The forces acting on the lung exert mechanical strain on these structures, leading to structural changes on all length scales during the breathing cycle [34]. The relationship between breathing state and dark-field signal is thus potentially very complex.

Furthermore, the mechanics of alveolar expansion are not fully understood, as methods for *in vivo* imaging of alveolar structures are limited: For example, optical coherence tomography (OCT) is able to resolve alveolar structures in the living lung, but only in depths up to 2–3 mm below the lung surface [35].

Multiple models for alveolar dynamics in the breathing cycle have been proposed, such as isotropic alveolar expansion/contraction, (de-)recruitment of alveoli, and alveolar shape change. Although there is evidence in favor of each model, the overall results suggest that a combination of isotropic expansion and alveolar shape changes is at work [34]. In particular, an *in vivo* time-resolved OCT imaging study on pigs found a predominance of uniformly expanding alveolar clusters [36].

Although calculations of X-ray dark-field signal from wave-optical simulation of simple models have been performed [37, 38], currently available data is too imprecise and contradictory to develop an accurate three-dimensional model of a breathing lung with sufficiently high resolution.

Thus, the present work seeks to identify individual contributions of microscopic and macroscopic changes in the lung to dark-field signal by experimental means, namely by correlating *in vivo* dark-field radiographs of the porcine lung to several macroscopic parameters retrieved by subsequent imaging of the pigs in a medical CT device. We used pigs due to the similar size and anatomy of porcine and human lungs. Pigs are also used as an animal model in translational respiratory research, partly for the same reasons [39].

Furthermore, motivated by results in [32], where dark-field CNR of pneumothoraxes were found to exceed those from conventional X-ray, we combine results from the correlation analysis with phantom measurements and calculations. This allows us to compare CNR of dark-field and conventional radiographs in the absence of anatomical structure, and thus, to examine the relative importance of various factors to CNR performance under realistic imaging conditions.

## Materials and methods

**Fig 1:**
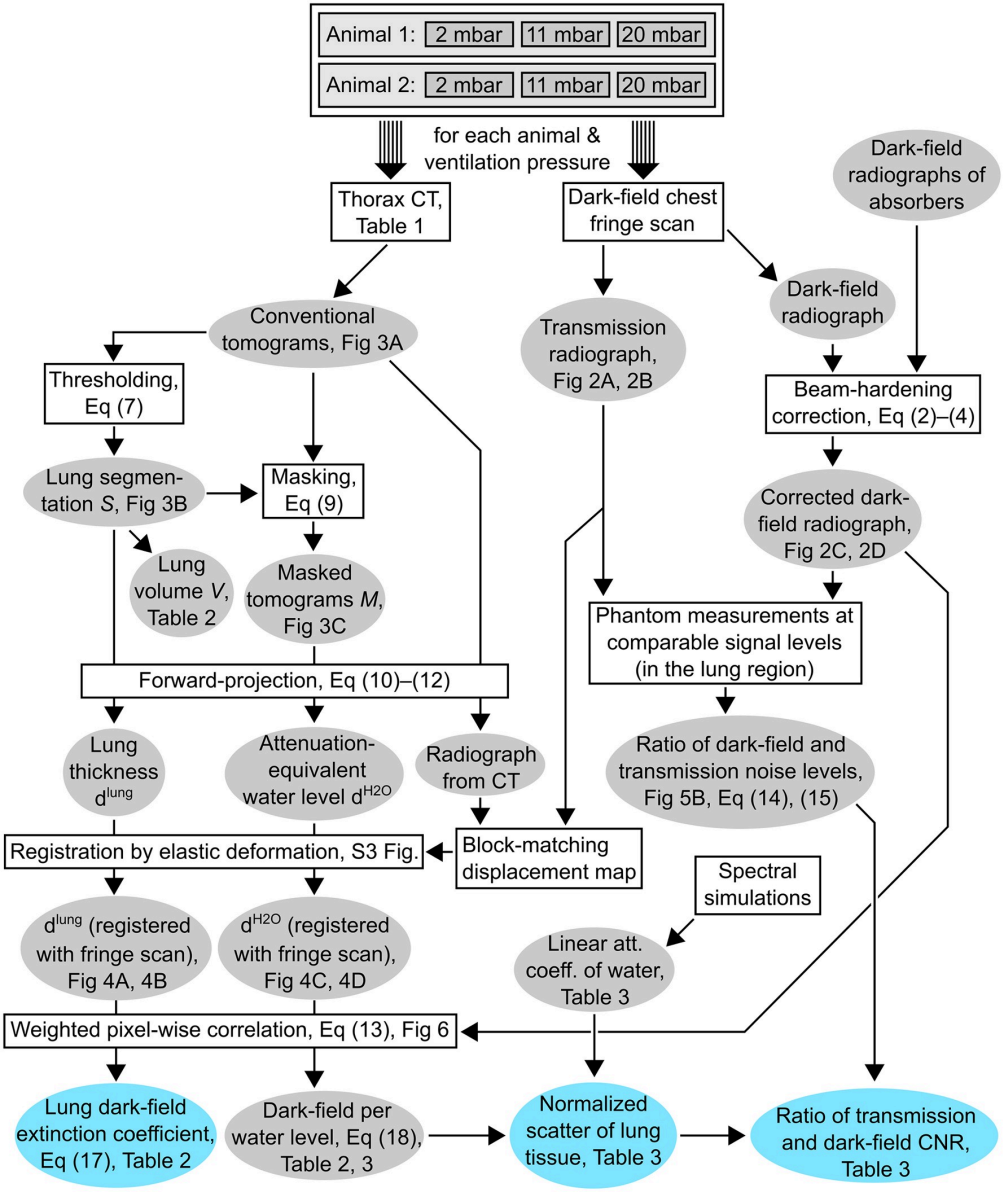
Flowchart of all processing and calculation steps. Datasets are shown as ellipses, operations performed on data are shown as rectangles. References to Equations, Figures and Tables are given where possible. Important findings are highlighted in color.

The goal of the calculation steps presented here is to combine the volumetric data obtained from the CT scans with the dark-field projection data acquired in the radiographic fringe-scanning acquisition, and to compare noise and CNR for the conventional and dark-field radiographs. The relationship between individual calculation steps and procedures, as well as their data output are summarized in Fig 1. Individual procedures or calculations are shown as rectangles, whereas data exchanged between them are shown as ellipses.

Dark-field radiographs retrieved by the fringe-scanning setup (cf. subsection “Imaging parameters”) are beam-hardening-corrected using previously acquired dark-field data of plastic absorbers of various heights (cf. subsection “Calculation of dark-field and attenuation values”). A number of phantom measurements were then performed to reproduce the range of dark-field and attenuation values achieved in the lung region to allow measurement of noise levels (cf. subsection “Noise estimation from phantom measurements and calculations”).

As detailed in the subsection “Lung segmentation and masking of CT data”, the lung is segmented from the CT data and a renormalized version of the CT data, which is masked to the lung volume, is calculated. These two datasets are then forward-projected, which yields maps of lung thickness and attenuation-equivalent water height. Their registration with data from the dark-field imaging setup is fine-tuned via application of an algorithm which applies elastic transformations to the thickness maps (cf. subsection “Forward-projection and registration with dark-field radiographs”).

Pixel-by-pixel correlation of the thickness and attenuation-equivalent water height maps with appropriate weighting (cf. subsection “Calculation of weighting coefficients for linear regression”) then yielded mean values for the lungs’ dark-field extinction coefficient, as well as a ratio of dark-field signal per water height. Dividing the latter quantity by the effective linear attenuation coefficient for water retrieved by spectral simulations (cf. subsection “Spectral simulation of attenuation coefficients for water”) retrieves the mean “normalized scatter” value of lung tissue.

Combining this result with the ratio of dark-field and attenuation noise levels (retrieved from calculations and phantom measurements) finally allows calculation of dark-field and attenuation contrast-to-noise ratio (CNR) for any given thickness difference of lung tissue.

### *In vivo* imaging procedure

Two German Landrace Hybrid pigs were used (wild type, Institute of Molecular Animal Breeding and Biotechnology, Ludwig Maximilian University Munich breeding facility; animal 1: male, animal 2: female; weight = 25 kg each; age 3 months; microbiological status not assessed). Animals were kept in conventional housing on continuous solid floor with straw bedding in age-matched groups. Animals were provided commercially-available pig feed and had unlimited access to water. Prior to the experiments, the animals were visually appraised by a veterinarian and no abnormalities were found.

The animals were sedated by intramuscular application of ketamine (Ursotamin^®^, Serumwerk Bernburg AG, Bernburg, Germany, 20 mg/kg) and azaperone (Stresnil^®^, Elanco Deutschland GmbH, Bad Homburg, Germany, 2 mg/kg). Anesthesia was continued by intravenous injection with propofol (Propofol 2%, MCT Fresenius, Fresenius Kabi AG, Langenhagen, Germany) using a syringe pump (Injectomat^®^MC Agilia, Fresenius Kabi AG, Langenhagen, Germany) with dose adjusted to effect. This method of anesthesia was applied to exclude spontaneous breathing during the experiment.

The animals were kept under automated ventilation throughout using an anesthesia machine (Fabius^®^ Tiro, Drägerwerk AG & Co. KGaA, Lübeck, Germany). Heart rate and oxygenation were monitored continuously using a pulse oximeter. Oxygen saturation did not fall below 90% at any point during the experiments. For imaging, ventilation was paused for the duration of dark-field radiographs and CT scans (max. 60 s at a time) with constant pressures of 2, 11, or 20 mbar in the airways, thus simulating expiration, intermediate inspiration, and full inspiration. All constant pressure values were set after achieving peak inspiratory pressure, i.e. during exhalation.

All animal procedures were performed with permission of the local regulatory authority, Regierung von Oberbayern (ROB), Sachgebiet 54, 80534 Munich, approval number AZ 55.2-1-54-2532-61-2015. The application was reviewed by the associated ethics committee according to §15 TSchG German Animal Welfare Law.

To terminate the experiment, the animals were euthanized under anesthesia by intravenous injection of T61^®^(Intervet GmbH, Unterschleissheim, Germany) according to the manufacturer’s instructions. The examinations were carried out in a non-survival experiment under continuous anesthesia to reduce burden on the animals. Sedation, anesthesia, imaging and termination were performed in two adjacent laboratory spaces. The experiments were conducted on a single day (7 AM until 3 PM) and were performed in sequence, i.e. the above procedure was begun for the second animal after euthanasia of the first animal. No randomization or blinding was performed.

### Imaging parameters

To determine a correlation between dark-field signal strength, airway pressure and projected thickness of the porcine lung, the two pigs were subsequently scanned at the dark-field scanning system and a medical 64-slice CT system (iCT SP, Koninklijke Philips N.V., Amsterdam, Netherlands). Two pigs were used instead of only one to allow quantifying the amount of variation of analysis results between animals. With each animal, three dark-field acquisitions and three helical CT scans were performed, at airway pressures of 2, 11, and 20 mbar. In order to allow for accurate registration of image data, care was taken to achieve a similar posture of the pigs and to precisely replicate the ventilation pressures in CT and dark-field measurements.

Detailed parameters of the dark-field scanning system were previously presented in [32]. To optimize dark-field image quality, however, the acquisition parameters were changed from the values given in [32]: The tube voltage was reduced from 70 to 60 kV and tube current was raised from 340 to 600 mA. Each part of the field of view received 25 X-ray pulses, resulting in a total scan time of 40 s. Despite the significant increase in tube current, dosimetric measurements under comparable conditions revealed only a moderate increase in entrance surface dose (ESD) from 720 to 913 μGy per scan. CT acquisition parameters are given in Table 1.

**Table 1:** Acquisition parameters for the CT scans. On each pig, three helical CT scans with the below acquisition and reconstruction parameters were performed at ventilation pressures of 2, 11, and 20 mbar. CTDI_vol_: volume CT dose index as calculated by the CT device (per scan). Relevant volume: smallest rectangular subset of the reconstructed volume containing the entire lung. Dimensions for volume and voxel size: lateral × dorsoventral × craniocaudal. Reconstruction was performed using filtered back projection with “Y-Sharp (YC)” convolution kernel. No noise reduction algorithms were applied.

	Tube voltage	Pitch	Scan time	CTDI_vol_	Relevant volume [mm^3^]	Voxel size [mm^3^]
Animal 1	120 kV	0.609	24.3 s	24.7 mGy	248 × 188 × 255	0.50 × 0.50 × 1.0
Animal 2	120 kV	0.609	25.7 s	24.3 mGy	264 × 182 × 275	0.42 × 0.42 × 1.0

### Calculation of dark-field and attenuation values

Given a mean X-ray flux *I*^r^ in the reference scan and *I* in the sample scan, sample transmittance in any given detector pixel (*x*, *y*) is defined as $T_{x,y}=I_{x,y}/I_{x,y}^{r}$. Similarly, with reference-scan visibility *V*^r^ and sample-scan visibility *V*, visibility reduction is given as $\nu_{x,y}=V_{x,y}/V_{x,y}^{r}$. Interferometric visibility is defined as

$$\begin{array}{c} V=\frac{I_{\text{max}}-I_{\text{min}}}{I_{\text{max}}+I_{\text{min}}}, \end{array}$$ (1)

with *I*_max_ (*I*_min_) being the highest (lowest) intensity achieved for all relative phases of analyzer grating and modulated intensity pattern. However, besides small-angle scatter due to electron density fluctuations in the sample, secondary effects such as beam-hardening and Compton scatter may lead to a reduction in visibility [40]. A correction was therefore applied to the acquired visibility reduction values, using a method closely resembling the one presented in [41]:

In a preceding calibration, visibility maps are acquired for different thicknesses *d*_1_, …, *d*_*N*_ of polyoxymethylene (POM). Due to the comparable X-ray interaction properties of POM and soft tissue, as well as identical placement of calibration material and actual sample in the beam, both the visibility-reducing effects of beam-hardening and Compton scatter are approximated by the calibration measurements. However, since POM is very homogeneous at the length scale of the setup’s correlation length, it does not cause significant small-angle scatter. The visibility reduction measured in POM is therefore only due to beam-hardening and Compton scatter.

By interpolation between visibilities $V_{k,x,y}^{\text{cal}}$ and transmittances $T_{k,x,y}^{\text{cal}}$ measured at POM heights *d*_*k*_, secondary visibility reduction *ν*^cal^ is interpreted as a function of transmittance, i.e.:

$$\begin{array}{c} \nu^{\text{cal}}\left(T_{k,x,y}^{\text{cal}}\right)=\frac{V_{k,x,y}^{\text{cal}}}{V_{x,y}^{r}},\;k=1,\ldots ,N. \end{array}$$ (2)

In order to limit acquisition time and data volume, calibration data was retrieved from a set of phase-stepping procedures at a single, central interferometer position. Furthermore, to account for spatial variations in grating parameters, the acquired values for *T*^cal^ and *V*^cal^ were averaged along the scanning direction *y*, over the full extent of the grating slot, yielding sets of values for each detector column *x* (orthogonal to *y*):

$$\begin{array}{c} \nu_{k,x}^{\text{cal}}={\left\langle \nu_{k,x,y}^{\text{cal}}\right\rangle }_{y},\;T_{k,x}^{\text{cal}}={\left\langle T_{k,x,y}^{\text{cal}}\right\rangle }_{y}. \end{array}$$ (3)

Using spline interpolation, functions $\nu_{x}^{\text{int}}\left(T\right)$ were calculated from these values, i.e. ${\left\{T_{k,x}^{\text{cal}},\nu_{k,x}^{\text{cal}}\right\}}_{k=1,\ldots ,N}$. These functions are evaluated for each pixel of the sample transmittance map *T*, yielding a pixel map of secondary visibility reduction factors $\nu_{x,y}^{\text{sec}}\equiv \nu_{x}^{\text{int}}\left(T_{x,y}\right)$.

Finally, to approximate the dark-field signal *D* in the absence of beam-hardening effects, the measured visibility reduction *ν* is divided by the evaluated map of secondary visibility reduction factors *ν*^sec^:

$$\begin{array}{c} D_{x,y}=\frac{\nu_{x,y}}{\nu_{x,y}^{\text{sec}}}. \end{array}$$ (4)

As beam-hardening was found to be negligible for the applied X-ray spectra and range of attenuation values, no corresponding correction was applied to *T*.

**Fig 2:**
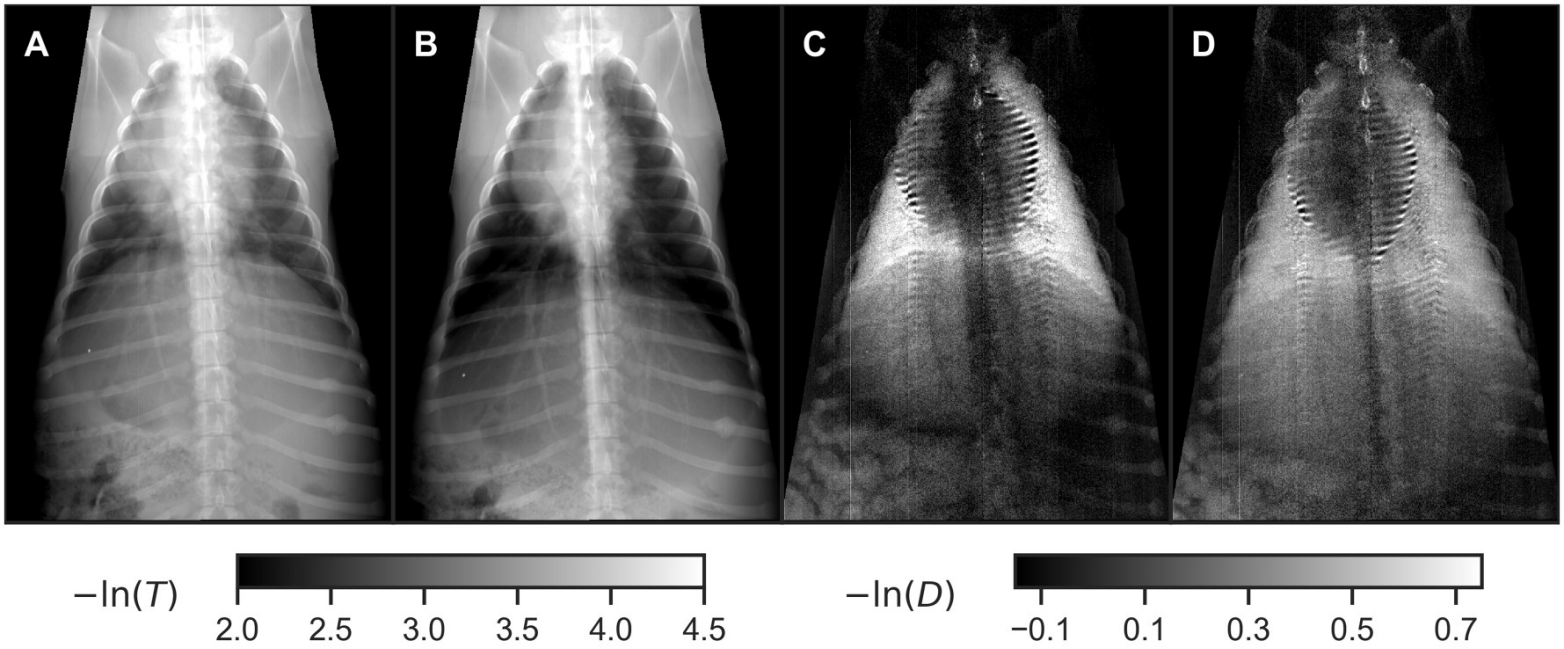
Transmission (A, B) and dark-field projections (C, D) from animal 1. (A), (C): 2 mbar ventilation pressure (simulated expiration). (B), (D): 20 mbar ventilation pressure (simulated inspiration).

Dark-field and attenuation images of one animal’s thorax at two ventilation pressures are shown in Fig 2. They are presented logarithmically, i.e. as −ln(*D*), and −ln(*T*), so that the signal values should be proportional to the thickness of a homogeneous absorber or scatterer, respectively.

### Effect of Compton scatter on the dark-field signal

At every point on the detector, measured intensity is the sum of Compton-scattered and non-scattered radiation: *I*_total_ = *I*_C_ + *I*_U_. *I*_U_ depends on relative grating shift *ϕ* via $I_{U}=\bar{I_{U}}\left[1+V\cos\left(\phi \right)\right]$, whereas Compton-scattered radiation is independent of *ϕ*, as it is incoherent and can thus not carry any visibility. Therefore,

$$\begin{array}{c} I_{\text{total}}=\bar{I_{U}}\left[1+V\cos\left(\phi \right)\right]+I_{C}. \end{array}$$ (5)

The visibility of *I*_total_ is thus $V_{C}=V{\left(1+I_{C}/\bar{I_{U}}\right)}^{-1}$. *ν* = *V*/*V*^r^ is also reduced by the factor ${\left(1+I_{C}/\bar{I_{U}}\right)}^{-1}$, since blank-scan visibilities *V*^r^ are measured without a sample and are thus unaffected by Compton scatter.

Data from measurements of *ν*^sec^ in Eq (4) also contain Compton-scattered radiation, but the ratio of intensities of Compton-scattered and non-scattered radiation ($I_{C}^{\text{sec}}/\bar{I_{U}^{\text{sec}}}$) may deviate due to the differing spatial distribution of material: Compton scattering occurs nearly isotropically, and scattered radiation detected at a certain position therefore originates from a large area of the sample. Calculation of *D* from *ν* and *ν*^sec^ via Eq (4) then yields

$$\begin{array}{c} \frac{D_{C}}{D}=\frac{1+I_{C}^{\text{sec}}\;/\;\bar{I_{U}^{\text{sec}}}}{1+I_{C}\;/\;\bar{I_{U}}}, \end{array}$$ (6)

with *D* the true and *D*_C_ the measured dark-field signals.

### Lung segmentation and masking of CT data

Lung segmentation was performed in the CT image volume via thresholding of Hounsfield unit (HU) values. For each CT volume, HU limits *L*^low^ and *L*^high^ were determined to bound HU values of all voxel inside the lung. For the scans with 20 and 11 mbar, *L*^low^ = −922 HU, *L*^high^ = −512 HU, for the scans with 2 mbar: *L*^low^ = −870 HU, *L*^high^ = −410 HU. For each data set, the corresponding segmentation volume *S* was defined as

$$\begin{array}{c} S_{x,y,z}=\{\begin{array}{cc} 1 & L^{\text{low}}<{\text{HU}}_{x,y,z}<L^{\text{high}}\\ 0 & \text{else} \end{array} \end{array}$$ (7)

for each voxel (*x*, *y*, *z*). Small holes in *S* were removed via binary closing with a (3 × 3 × 3) voxel kernel. The determined thresholds were verified by comparison of the segmented volume with the lung boundaries visible in the original dataset.

Furthermore, an attenuation coefficient relative to water was calculated from the HU values via

$$\begin{array}{c} \frac{\mu }{\mu^{H2O}}=\frac{\text{HU}-{\text{HU}}^{\text{air}}}{{\text{HU}}^{H2O}-{\text{HU}}^{\text{air}}}, \end{array}$$ (8)

where HU^H2O^ = 0 and HU^air^ = −1000 are the HU values of water and air, respectively. To be able to isolate the portion of the attenuation signal originating from the lung, the volume of relative attenuation values was “masked” by the segmentation *S*, yielding the dataset *M*:

$$\begin{array}{c} M_{x,y,z}=S_{x,y,z}\cdot {\left(\frac{\mu }{\mu^{H2O}}\right)}_{x,y,z} \end{array}$$ (9)

**Fig 3:**
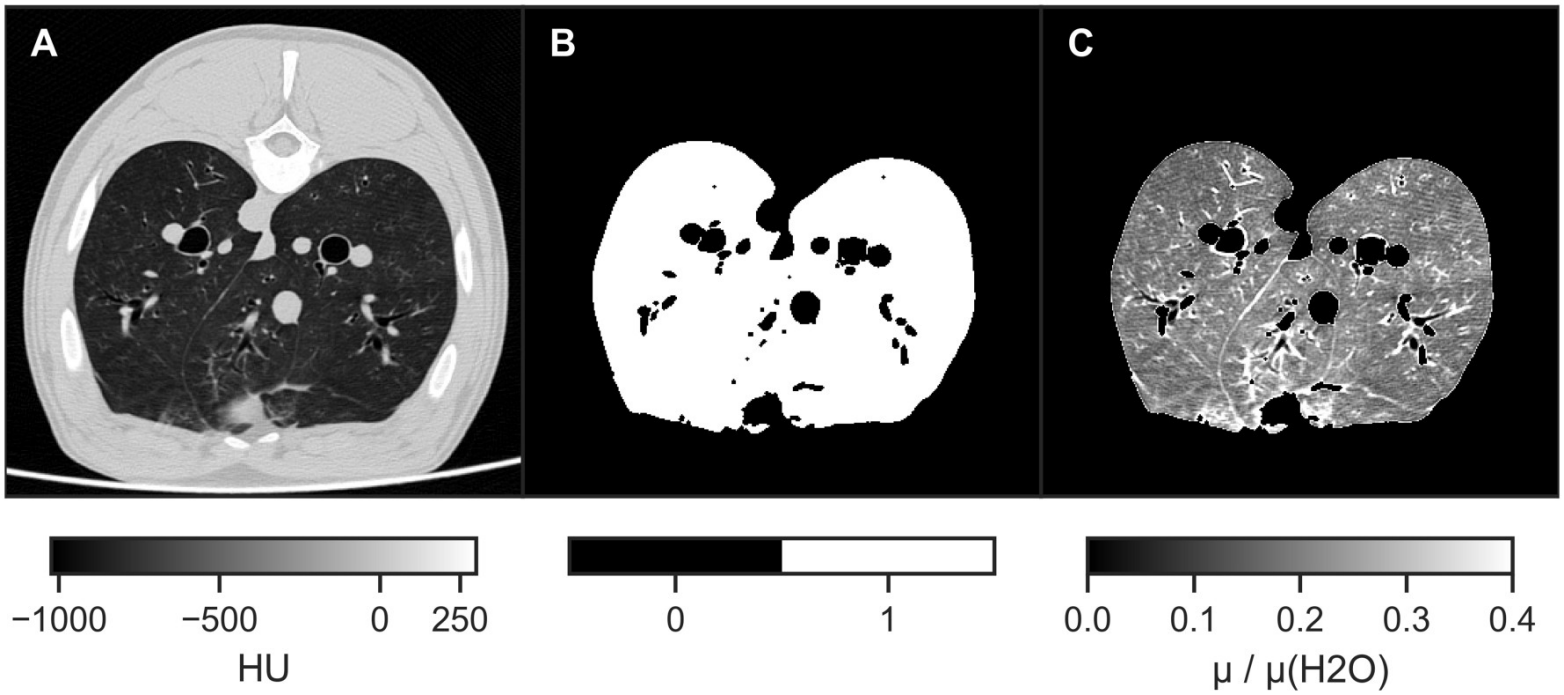
CT slice and lung segmentation / masking (animal 2). (A) Slice from reconstruction of one of the CT scans (20 mbar). (B) Segmentation *S* of the lung via thresholding and binary closing as defined in Eq (7). Interior of bronchi and more strongly attenuating tissues (HU ≈ 0) are excluded. (C) Masked volume of relative attenuation values *M*, as defined in Eq (9).

The effect of segmentation and masking on one example CT slice are illustrated in Fig 3.

### Forward-projection and registration with dark-field radiographs

**Fig 4:**
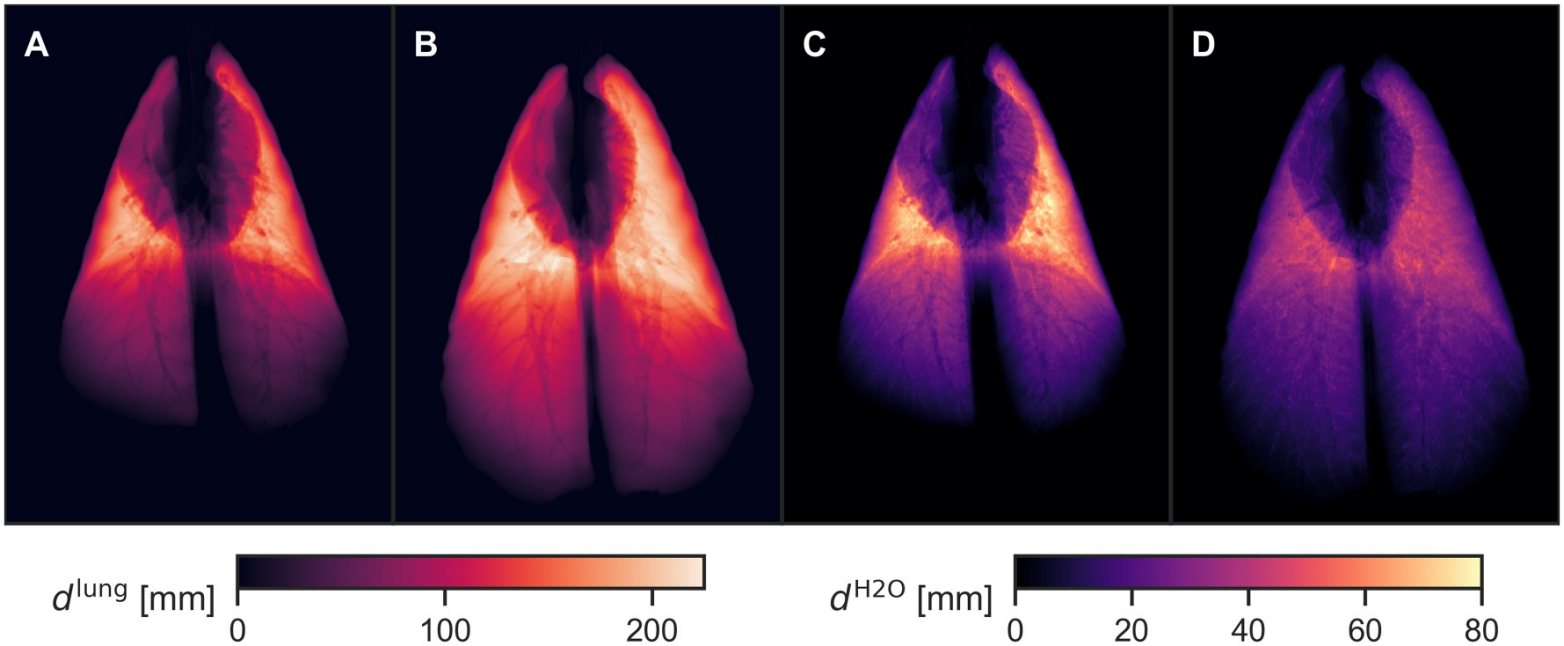
Forward-projections of segmented and masked CT data (animal 1). (A, B): Maps of projected lung thickness *d*^lung^ [forward-projections of segmentation volumes *S*, cf. Eq (10)], for 2 and 20 mbar ventilation pressure. (C, D): Maps of attenuation-equivalent water level *d*^H2O^ [forward-projections of relative attenuation volumes *M*, cf. Eq (12)], for 2 and 20 mbar ventilation pressure. Larger airways are visible in both types of maps, but density variations of lung parenchyma are much more apparent in maps of *d*^H2O^.

Forward-projection of the binary segmentation volume *S* produces a map of its thickness *d*^lung^ in the direction of projection:

$$\begin{array}{c} d_{x,y}^{\text{lung}}=\int_{R(x,y)}S\left(\vec{r}\right)d\vec{r}, \end{array}$$ (10)

with *S* from Eq (7) interpolated from the discrete to the continuous domain, (*x*, *y*) a position in the detector plane, and *R*(*x*, *y*) a straight line from the source to position (*x*, *y*) on the detector. This quantity is insensitive to density variations within the lung volume, such as those occurring between different inspiration states. An example is shown in Fig 4A and 4B.

Forward-projection of the masked image of relative attenuation coefficients *M* yields a height of an equivalently absorbing layer of water. This can be seen by equating an arbitrary projection integral with that of a given water layer and solving for its height *d*^H2O^:

$$\begin{array}{cc} -\ln T_{x,y} & =\int_{R(x,y)}S\left(\vec{r}\right)\mu \left(\vec{r}\right)\;d\vec{r}\overset{!}{=}\mu^{H2O}\cdot d_{x,y}^{H2O} \end{array}$$ (11)

$$\begin{array}{cc} \Rightarrow d_{x,y}^{H2O} & =\int_{R(x,y)}S\left(\vec{r}\right)\cdot \frac{\mu (\vec{r})}{\mu^{H2O}}\;d\vec{r}\overset{\left(9\right)}{=}\int_{R(x,y)}M\left(\vec{r}\right)\;d\vec{r}, \end{array}$$ (12)

with *S*, *μ*/*μ*_H2O_, and *M* from Eqs (7)–(9) interpolated from the discrete to the continuous domain to allow integration. The maps of *d*^H2O^ values retrieved from two of the CT scans are shown in Fig 4C and 4D. For the sake of brevity, we omit the (*x*, *y*) pixel subscripts in the following. We use this quantity to interrelate the transmittance values obtained by the dark-field setup and the CT device: Since the two setups use very different X-ray spectra and detectors with differing spectral quantum efficiencies, the retrieved transmittance values are not easily comparable. However, the attenuation spectra of soft tissue and water are very similar in the range of medically-used X-ray energies, which means that the height level *d*^H2O^ of water, which is equivalent in attenuation to a given amount of lung, is approximately independent of acquisition parameters. Conversion of *d*^H2O^ to logarithmic transmittance values −ln(*T*) measured at the dark-field setup is then possible by simulating the X-ray spectrum as seen by the detector and calculating the effective linear attenuation coefficient of water *μ*^H2O^ for this spectrum.

Cone-beam forward-projection [interpolation of *S*, *M* to the continuous domain and calculation of the integrals in Eqs (10) and (12)] was performed using a reconstruction software package developed at the Chair of Biomedical Physics, TUM. Projection geometry parameters were selected to match the true geometry of the dark-field setup (as given in [32]), and projection angles were manually adjusted to optimize registration of the results with data from the dark-field setup.

Lastly, the plugin bUnwarpJ [42] for the image processing software Fiji [43] was used to apply elastic deformations on the projected CT data to account for deviations between the positioning of the pigs in CT and dark-field setup, and thus further improve registration with the dark-field radiographs. In a first step, feature pairs from projected CT data and the corresponding attenuation image from the dark-field setup were found by applying a block-matching algorithm in Fiji. These pairs of image coordinates were then used as input for bUnwarpJ, yielding a slightly distorted version of the projected CT data. Visual inspection showed nearly perfect registration of the image pairs, enabling more accurate calculation of pixel-for-pixel correlations. Magnitude and shape of the introduced distortion are visualized in S3 Fig for one of the six dataset pairs.

### Calculation of weighting coefficients for linear regression

We performed linear regression between −ln(*D*) and *d*^lung^, as well as between −ln(*D*) and *d*^H2O^. To account for variable noise levels across different regions in the image, weighting of individual data points was applied in accordance with a simplified version of the model for the propagation of shot noise to the dark-field modality, as presented in [44]. Specifically, we used the equation

$$\begin{array}{c} {\sigma (D)}^{2}\propto \frac{D^{2}}{{\left(V^{r}\right)}^{2}I^{r}}[{\left(V^{r}\right)}^{2}(1+\frac{1}{T})+2(1+\frac{1}{TD^{2}})], \end{array}$$ (13)

where *V*^r^ and *I*^r^ are visibility and detected photon counts in the flat-field acquisition. All quantities in Eq (13) are to be understood as functions of pixel indices (*x*, *y*). Fit weights were set as *w* ∝ *σ*(*D*)^−2^. Simplifications with respect to [44] are in assuming a constant gain for sample and blank scan acquisition, and a proportionality between *I*^r^ and the signal of the (energy-integrating) detector. Furthermore, the cited equation assumes a phase-stepping acquisition of the images, whereas the presented images were acquired with a fringe-scanning acquisition. Since the model for signal extraction is similar for both acquisition schemes (see e.g. [26]), Eq (13) is reasonably well applicable here.

### Spectral simulation of attenuation coefficients for water

The MATLAB package spektr 3.0 [45] was used to simulate X-ray spectra for two imaging situations, namely the presented three-grating setup operated at 60 kV, and a system without gratings, operated at 125 kV (simulating a setup for conventional thorax imaging, as they are typically operated at 125 to 150 kV [46]). The software calculates spectral X-ray flux histograms of tungsten anode X-ray tubes with 1 keV bins.

For both scenarios, tube filtration of 2.5 mm Al was assumed and the spectral fraction of absorbed X-ray flux in a 600 μm detector layer of CsI scintillation material (as used in the setup’s real detector) was then calculated. For the first scenario, filtering due to the three gratings is also taken into account. The energy-integrating property of the detector signal in a flat-panel detector was modeled by multiplying each energy bin of the spectral absorbed flux with the bin’s photon energy before adding up all the products to calculate the detector signal.

An effective linear attenuation coefficient for water was calculated for each scenario by including attenuation due to various heights of water (up to 20 cm) in the spectral calculation, and performing linear regression of logarithmic transmittance with respect to water height. Nonlinearities due to beam-hardening were found to be negligible.

### Noise estimation from phantom measurements and calculations

For the estimation of dark-field and transmission noise levels, a phantom was constructed from polyoxymethylene (POM, Hans-Erich Gemmel & Co. GmbH, Berlin, Germany) and chloroprene (CR-L, W. Köpp GmbH & Co. KG, Aachen, Germany). Four thickness combinations (*d*^POM^, *d*^CRL^) were measured to approximate dark-field and attenuation levels achieved in different regions of the pig scans: (6.4 cm, 3.0 cm), (9.6 cm, 2.0 cm), (9.6 cm, 1.0 cm), (12.8 cm, 0 cm).

Noise levels were also calculated from dark-field and transmission signal levels, adapting results from [44]: It was assumed that detector gain is identical for flat-field and sample images, and that standard deviations of logarithmic modalities can be approximated by

$$\begin{array}{c} \sigma \left(\ln X\right)\approx \sigma \left(X\right)\cdot {\frac{\partial \ln x}{\partial x}|}_{\left\langle X\right\rangle }=\frac{\sigma (X)}{\left\langle X\right\rangle }\;\left(X=D,T\right). \end{array}$$ (14)

We thus find that

$$\begin{array}{c} \frac{\sigma (\ln D)}{\sigma (\ln T)}\approx \sqrt{1+\frac{2}{{\left(V^{r}\right)}^{2}}\frac{1+\frac{1}{TD^{2}}}{1+\frac{1}{T}}}, \end{array}$$ (15)

where *V*^r^ is the blank scan visibility, for which we assumed the mean measured value of 0.365.

### Calculation of attenuation signal fraction due to the lung

Multiplying the water-equivalent map of the pig lung (*d*^H2O^, cf. Fig 4C and 4D) with the simulated attenuation coefficient of water in the dark-field setup at 60 kV, $\mu_{\text{eff},60}^{\text{H2O}}$, yields a map of the attenuation signal due to the lung alone. This can then be related to the corresponding attenuation image −ln *T* of the whole thorax from the dark-field setup (also acquired at 60 kV, cf. Fig 2A and 2B) to yield a pixel map of the fraction *f* of attenuation signal due to the lung:

$$\begin{array}{c} f=\frac{\ln T^{\text{lung}}}{\ln T}=-\frac{d^{\text{H}2\text{O}}\mu_{\text{eff},60}^{\text{H2O}}}{\ln T} \end{array}$$ (16)

## Results

### Magnitude of correction procedures

**Fig 5:**
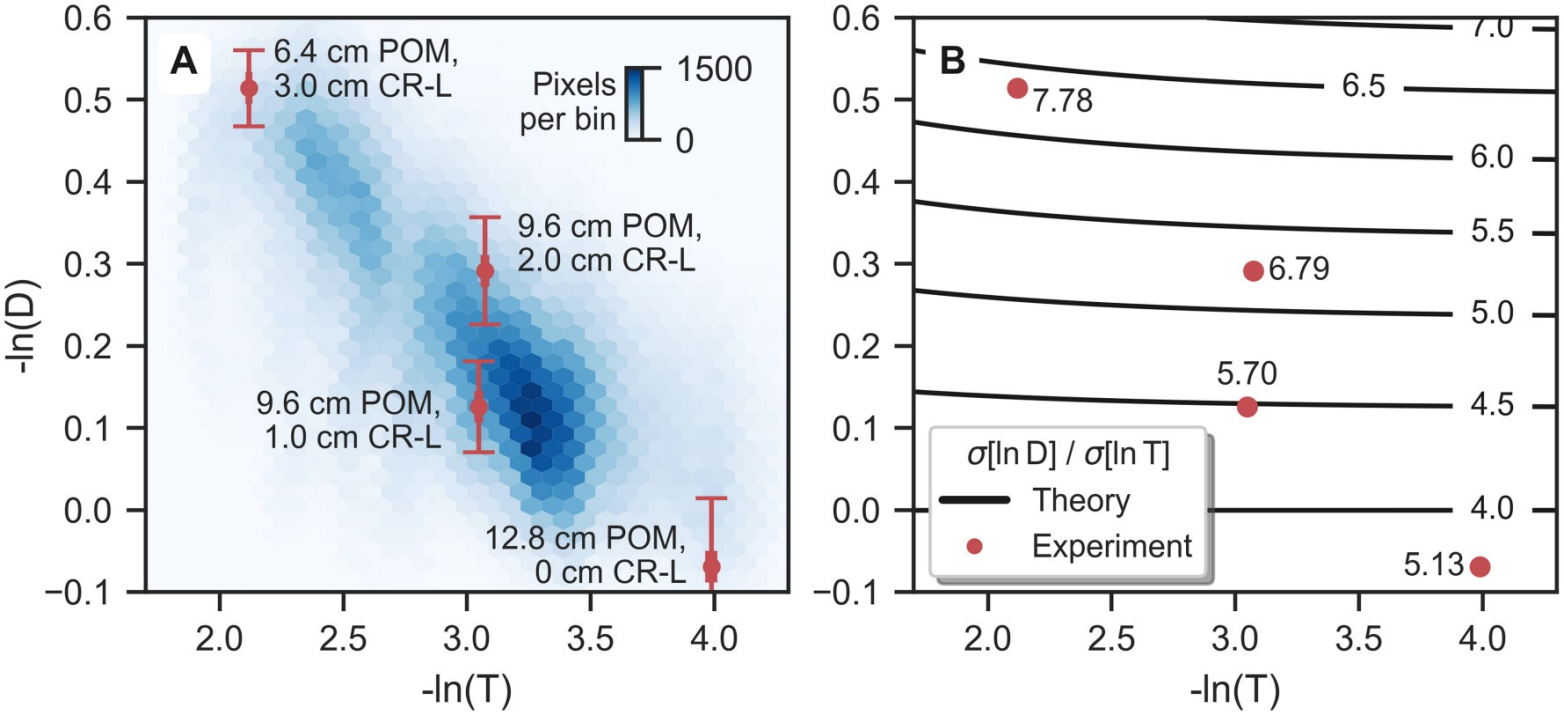
Dark-field and attenuation noise levels in the lung area of one pig scan. (A) Bivariate histogram of signal values (−ln *D*, −ln *T*) from *in vivo* dark-field radiography of the pig thorax (animal 1, 20 mbar). Pixels where *d*^lung^ > 3 mm are included. Four phantom measurements were performed at similar signal levels to determine signal standard deviations *σ* (red markers and error bars), *σ*(ln *T*) values are very small. (B) Ratio of standard deviations *σ*(ln *D*)/*σ*(ln *T*) from the phantom measurements (red markers), and theoretically calculated values (Eq (15), black contour lines), displayed as a function of signal levels.

Beam-hardening correction of dark-field radiographs has a significant influence on signal levels: The logarithmic secondary visibility reduction −ln *ν*^sec^ retrieved from calibration measurements was approximately 10% of logarithmic transmittance −ln *T*. Given that the latter ranges between 2.0 and 4.0 in the lung area of the radiographs (Fig 5A), the beam-hardening-corrected dark-field values −ln *D* are about 0.2 to 0.4 below the measured values −ln *ν*, namely in a range between −0.1 and 0.6 (Fig 5A).

The method used for the registration of forward-projected CT data onto the corresponding dark-field radiographs (spline-based elastic deformation) implies that the input data is being slightly distorted. Direction and magnitude of this distortion are illustrated for one pair of images in S3 Fig. Displacement of lung features in this dataset ranged between 1.1 and 9.5 mm, with a mean displacement of 6.1 mm.

### Correlation between ventilation pressure and signal level

For all six pairs of measurements (two pigs, three values of ventilation pressure), the correlations of −ln *D* with both projected lung thickness *d*^lung^ and transmission-equivalent water level *d*^H2O^ were analyzed. In the outermost periphery of the lung, minor discrepancies in registration lead to large errors due to the sharp transition between lung and the surrounding tissue. This region, here defined as all pixels with 0 < *d*^lung^ < 3 mm, was thus excluded from the analysis.

**Fig 6:**
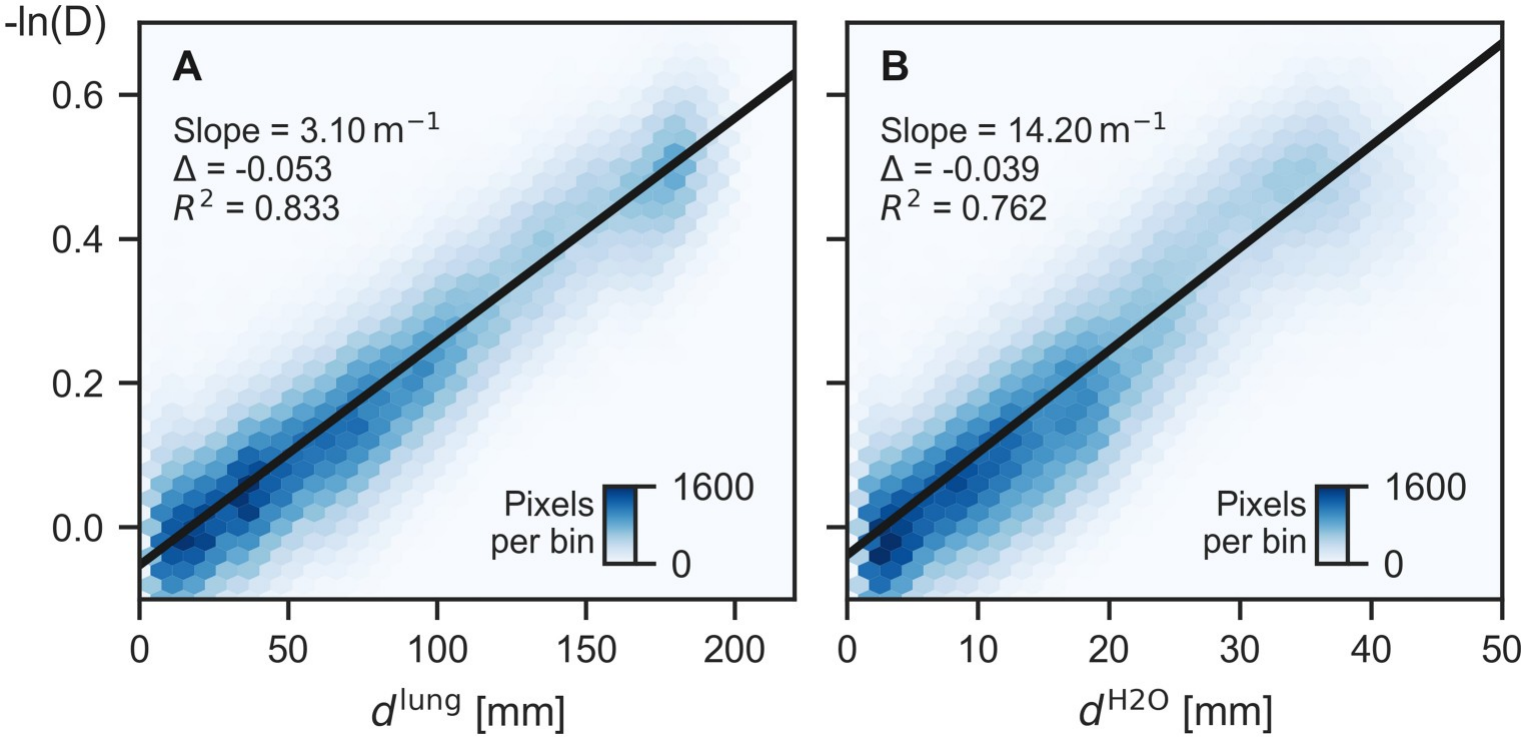
Bivariate histograms of logarithmic dark-field signal vs. *d*^lung^ (A) and *d*^H2O^ (B). Results from linear regression are superimposed. The imaging data from simulated inspiration (20 mbar ventilation pressure, animal 2) is shown here. Regression results for all measurements are compiled in Table 2 and equivalent bivariate histograms for all measurements are shown in S1 Fig.

To visualize the correlation of −ln *D* with *d*^lung^ and *d*^H2O^, we avoided scatter plots because they are unable to visualize details in regions of high plot point density. As shown in Fig 6, we instead present bivariate histograms, where local average point density, i.e. the number of image pixels per hexagonal bin, is color-coded.

The degree of linear correlation between −ln *D* and *d*^lung^, *d*^H2O^ was quantified by linear regression with weighting according to Eq (13). A superposition of regression results onto the corresponding bivariate histograms is shown in Fig 6. The corresponding graphs for all measurements are shown in S1 Fig.

The slope of the linear regression with respect to *d*^lung^ is equivalent to the “dark-field extinction coefficient” (the dark-field equivalent to the linear attenuation coefficient *μ*, named *μ*_d_ by Lynch et al. in [8]), averaged over the entire lung:

$$\begin{array}{c} -\frac{\partial (\ln D)}{\partial d^{\text{lung}}}={\left\langle \mu_{d}\right\rangle }_{\text{lung}}. \end{array}$$ (17)

It decreases significantly with rising ventilation pressure (by −33 and −42% from 2 to 20 mbar), cf. Table 2.

**Table 2:** Results from regression between −ln *D* and projected lung thickness *d*^lung^, as well as between −ln *D* and *d*^H2O^. −*∂*(ln *D*)/*∂d*^lung^ is a global average of the lungs’ dark-field extinction coefficient, cf. Eq (17). The corresponding histograms and regression curves are shown in S1 Fig. Lung volumes were retrieved from the segmentation *S*, cf. Eq (7).

	Animal 1			Animal 2		
Pressure [mbar]	2	11	20	2	11	20
−*∂*(ln *D*)/*∂d*^lung^[m^−1^]	**4.11**	**2.74**	**2.38**	**4.62**	**3.48**	**3.10**
Intercept Δ	-0.068	-0.047	-0.003	-0.083	-0.067	-0.053
*R*^2^ of fit	0.766	0.716	0.705	0.853	0.836	0.833
−*∂*(ln *D*)/*∂d*^H2O^[m^−1^]	**10.68**	**11.11**	**10.82**	**12.86**	**13.70**	**14.20**
Intercept Δ	-0.048	-0.021	0.025	-0.079	-0.059	-0.039
*R*^2^ of fit	0.737	0.665	0.646	0.819	0.783	0.762
Lung volume [l]	0.76	1.20	1.37	0.78	1.06	1.24

**Fig 7:**
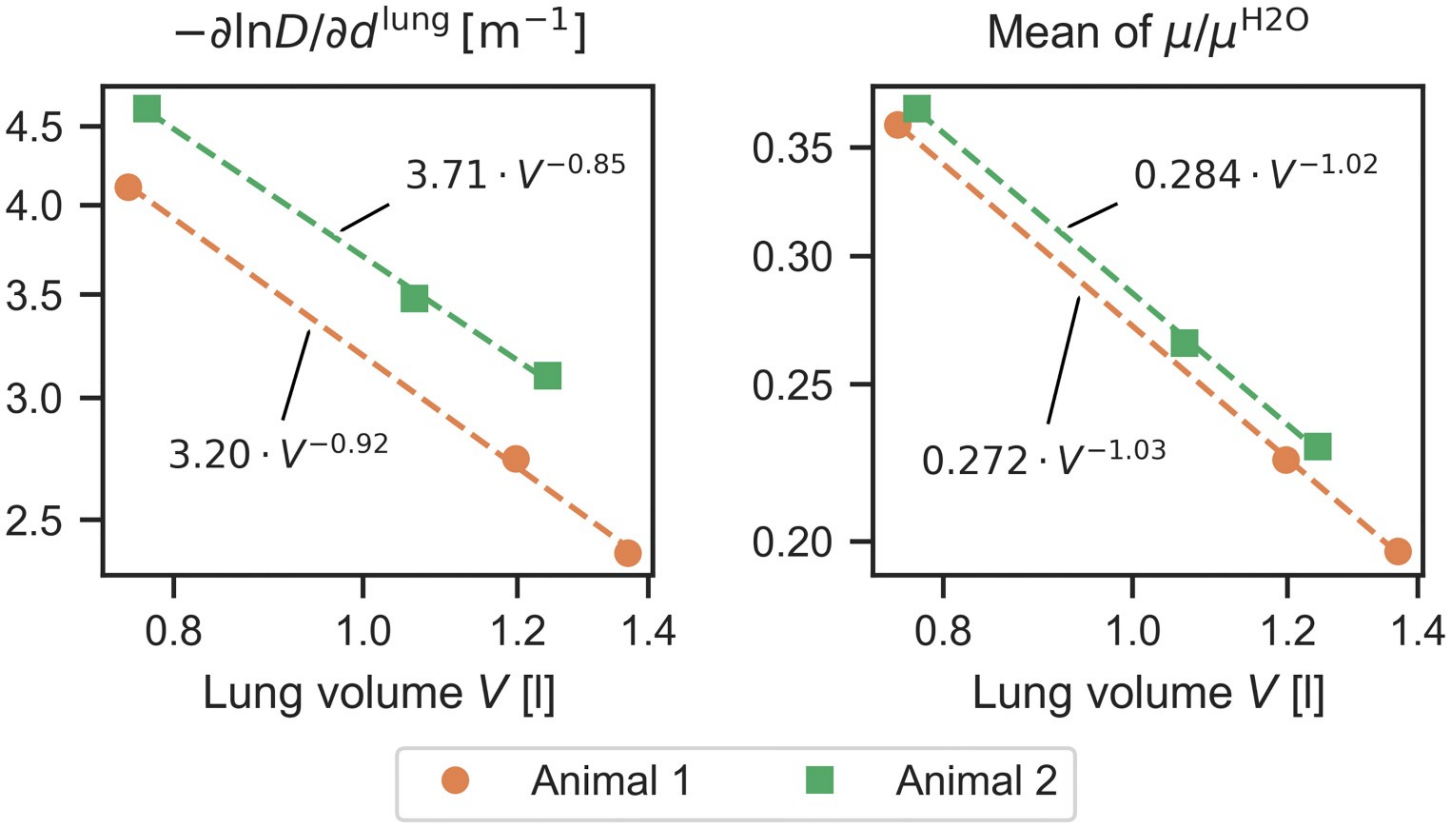
Dependence of lungs’ average dark-field extinction coefficient (−*∂* ln *D*/*∂d*^lung^) and relative linear attenuation coefficient (*μ*/*μ*^H2O^) on lung volume. The three data points correspond to 2, 11, and 20 mbar, respectively. Axes are scaled logarithmically. Mean linear attenuation coefficients are retrieved from HU values in lung segmentation and are inversely proportional to lung volume. Dark-field extinction coefficients decrease more slowly (exponents greater than −1), which could e.g. be due to alveolar recruitment at higher ventilation pressures.

The observed decrease, both in −*∂*(ln *D*)/*∂d*^lung^, as well as in the lungs’ mean linear attenuation coefficient *μ* for rising pressure is related to the simultaneous increase of lung volume. As *μ* is proportional to mass density for a given material, the integral of *μ* over the entire lung volume should be independent of ventilation pressure. In other words, the lungs’ mean linear attenuation coefficient should be inversely proportional to lung volume. To verify this, we determined these quantities from the segmented and masked CT volumes (*S*, *M*) and plotted *μ*/*μ*^H2O^, as well as −*∂*(ln *D*)/*∂d*^lung^ as a function of lung volume and applied regression of a power-law function *y* = *aV*^*b*^ (Fig 7). While an inversely proportional relationship is observed for *μ*/*μ*^H2O^ (*b* ≈ −1), this is not quite the case for −*∂*(ln *D*)/*∂d*^lung^, where we find exponents *b* > −1.

On the other hand, dark-field signal per absorption-equivalent water height is equivalent to the ratio of the lung’s mean dark-field extinction coefficient and linear attenuation coefficient normalized to water:

$$\begin{array}{c} -\frac{\partial (\ln D)}{\partial d^{H2O}}=\frac{{\left\langle \mu_{d}\right\rangle }_{\text{lung}}}{{\left\langle \mu /\mu^{H2O}\right\rangle }_{\text{lung}}}=\underset{\text{Norm}.\;\text{scatter}}{\underset{︸}{\frac{{\left\langle \mu_{d}\right\rangle }_{\text{lung}}}{{\left\langle \mu \right\rangle }_{\text{lung}}}}}\cdot \;\mu_{H2O}. \end{array}$$ (18)

*μ*_d_/*μ* has been called the “normalized scatter” signal [19]. −*∂*(ln *D*)/*∂d*^H2O^ is thus proportional to an average normalized scatter of the entire lung (excluding surrounding tissues).

The results shown in Fig 7 suggest that for increasing ventilation pressure, *μ*_d_ decreases more slowly than *μ*, and thus, an increase of −*∂*(ln *D*)/*∂d*^H2O^ should be observed. This is indeed the case: The values for 11 mbar (20 mbar) exceed those for 2 mbar by 4% (1.3%) for animal 1, and by 6.5% (10%) for animal 2. Slopes and coefficients of determination for each regression analysis, as well as lung volumes for all measurements are summarized in Table 2.

To estimate the lungs’ normalized scatter signal from −*∂*(ln *D*)/*∂d*^H2O^, the effective linear attenuation coefficient of water must be determined [cf. Eq (18)], which was done by simulation. We present calculated values for $\mu_{\text{eff}}^{\text{H2O}}$ in two imaging scenarios (cf. Methods): The first ($\mu_{\text{eff},60}^{\text{H2O}}$) is valid for the presented dark-field setup when operated at 60 kV (and thus relevant for the calculation of normalized scatter), the second ($\mu_{\text{eff},125}^{\text{H2O}}$) is achieved in a conventional radiographic system operated at 125 kV, and is provided only for comparison. We find normalized scatter values between 0.4 and 0.5 (cf. Table 3). These results will be revisited in the analysis of noise levels further below.

**Table 3:** Calculation of dark-field-to-attenuation CNR ratios of *in vivo* porcine lung tissue. $\mu_{\text{eff},60}^{\text{H2O}}$, $\mu_{\text{eff},125}^{\text{H2O}}$: effective linear attenuation coefficients of water for the dark-field setup at 60 kV and a setup without gratings at 125 kV. −*∂*(ln *D*)/*∂d*^H2O^: slopes from linear regression of −ln *D* w.r.t. *d*^H2O^, cf. Table 2. Normalized scatter values are calculated using Eq (18). Via Eq (23), we join them with the ratio of standard deviations for realistic signal levels (cf. Fig 5B) to determine ratios of dark-field and transmission CNR due to small differences in lung thickness.

$\mu_{\text{eff},60}^{\text{H2O}}$	25.42 m^−1^	
$\mu_{\text{eff},125}^{\text{H2O}}$	21.07 m^−1^	
	Animal 1	Animal 2
−*∂*(ln *D*)/*∂d*^H2O^	(10.68 … 11.11)m^−1^	(12.86 … 14.20)m^−1^
Normalized scatter	0.420 … 0.437	0.506 … 0.559
*σ*(ln *D*)/*σ*(ln *T*)	5.1 … 7.8	
CNR(ln *D*)/CNR(ln *T*)	0.054 … 0.11	

It is apparent that a vertical offset (i.e. nonzero vertical axis intercept) is present in these histograms, which varies between ventilation pressures. We believe that this is primarily due to a deviation between the fraction of Compton-scattered and non-scattered radiation in the sample scan, when compared to the beam-hardening measurement ($I_{C}/\bar{I_{U}}$ and $I_{C}^{\text{sec}}/\bar{I_{U}^{\text{sec}}}$ in Eq (6), respectively):

$I_{C}^{\text{sec}}/\bar{I_{U}^{\text{sec}}}$ is likely to be very nearly position-independent, since the beam-hardening measurement is performed with homogeneous slabs of POM. We think that $I_{C}/\bar{I_{U}}$ can also be assumed to be approximately constant in the area of the lung, since Compton scatter is nearly isotropic and the sample is quite distant from the detector (> 30 cm). Taking the logarithm of Eq (6) and solving for −ln *D*_C_, which is the quantity on the vertical axis in Fig 6 and S1 Fig, shows it is the sum of the true signal −ln *D* (which we assume to increase proportionally with *d*^lung^ and *d*^H2O^) and a second term Δ, which should be approximately constant in the whole area of the lung:

$$\begin{array}{c} -\ln\left(D_{C}\right)=\underset{\propto \;d^{\text{lung}},\;d^{H2O}}{\underset{︸}{-\ln D}}-\ln(\frac{D_{C}}{D})\overset{\left(6\right)}{=}-\ln D+\underset{\Delta }{\underset{︸}{\ln(\frac{1+I_{C}\;/\;\bar{I_{U}}}{1+I_{C}^{\text{sec}}\;/\;\bar{I_{U}^{\text{sec}}}})}} \end{array}$$ (19)

For $I_{C}/\bar{I_{U}},I_{C}^{\text{sec}}/\bar{I_{U}^{\text{sec}}}⪡1$, $\Delta \approx I_{C}/\bar{I_{U}}-I_{C}^{\text{sec}}/\bar{I_{U}^{\text{sec}}}$. Values for Δ achieved from linear regression are given in Table 2.

### Comparison of noise and CNR for dark-field and attenuation radiography

Direct estimation of noise levels from the *in vivo* scans is difficult due to the high spatial variability of both attenuation and dark-field signals. Therefore, dark-field scans of a simple phantom were performed at identical acquisition parameters.

POM was used to simulate attenuation due to soft tissue and fat, whereas the dark-field signal due to the lung was simulated by chloroprene sheets. The amount of phantom material was selected such that similar levels of dark-field and attenuation signal were achieved. Due to the high uniformity of the materials across the field of view, standard deviations of ln *D* and ln *T* could be estimated in manually-defined regions of interest of the phantom measurements.

The phantom materials were selected due to their similarity in spectral X-ray interaction parameters: The linear attenuation coefficients of POM and soft tissue as a function of X-ray energy are approximately proportional for medically-used X-ray energies, and spectrally-resolved X-ray dark-field measurements have shown a comparable energy-dependence as ventilated porcine *ex situ* lung tissue up to about 70 keV (unpublished work). To ensure comparability of results, the correction for secondary visibility reduction effects was also applied for the phantom scans.

In order to illustrate the achieved range of signal values in the *in vivo* scans, a bivariate histogram of dark-field and attenuation values from the lung-covered area in one of the measurements is presented in Fig 5A. Four phantom thickness combinations were selected to characterize this range. Mean and standard deviations from these measurements are shown in red (relative errors in −ln *T* are very small).

The measured ratios of dark-field and transmission standard deviations are shown side-by-side with theoretically calculated values in Fig 5B (see Methods for calculation).

The experimentally determined values exceed the theoretical values, which may be due to the presence of unresolvable structures within the chloroprene sheets, generating a noise-like pattern (“structural noise”). Assuming statistical independence of shot noise and structural noise, their variances can be added:

$$\begin{array}{c} \sigma^{2}=\sigma_{\text{shot}}^{2}+\sigma_{\text{struct}}^{2}. \end{array}$$ (20)

For the ratios of dark-field and transmission variances, it thus follows that

$$\begin{array}{c} \frac{\sigma (\ln D)}{\sigma (\ln T)}=\sqrt{\frac{\sigma_{\text{shot}}^{2}\left(\ln D\right)+\sigma_{\text{struct}}^{2}\left(\ln D\right)}{\sigma_{\text{shot}}^{2}\left(\ln T\right)+\sigma_{\text{struct}}^{2}\left(\ln T\right)}}=\frac{\sigma_{\text{shot}}\left(\ln D\right)}{\sigma_{\text{shot}}\left(\ln T\right)}\sqrt{\frac{1+{\tilde{\sigma }}^{2}\left(\ln D\right)}{1+{\tilde{\sigma }}^{2}\left(\ln T\right)}}, \end{array}$$ (21)

where $\tilde{\sigma }=\sigma_{\text{struct}}/\sigma_{\text{shot}}$. Identifying *σ*(ln *D*)/*σ*(ln *T*) with the measured and *σ*_shot_(ln *D*)/*σ*_shot_(ln *T*) with the theoretical noise levels ratios, it can be seen that their quotient is equal to $\sqrt{\left(1+{\tilde{\sigma }}_{D}^{2}\right)/\left(1+{\tilde{\sigma }}_{T}^{2}\right)}$. Since that quotient is greater than 1 for all measured points (cf. Fig 5B), it must follow that ${\tilde{\sigma }}_{D}>{\tilde{\sigma }}_{T}$, i.e. “structural” noise is more important compared to shot noise in the dark-field images than in the conventional images of the phantom. This is not too surprising: The absorbing plastic in the phantom is likely very homogeneous, whereas the ability of the chloroprene sheets to generate dark-field signal is intrinsically dependent on the presence of microscopic fluctuations of electron density. For chloroprene, and probably most dark-field-active materials, these fluctuations also extend to much greater length scales (i.e., near the effective pixel size), and thus intrinsically create some level of structural noise. We therefore believe that pure (spatial) dark-field shot noise will rarely be observed, even in measurements of outwardly uniform dark-field phantom materials.

Finally, the estimates for noise level ratios can be combined with the previously determined normalized scatter estimates to determine a combined quantity: Given two regions *a*, *b* in a thorax dark-field radiograph, with different (but otherwise arbitrary) levels of lung material in each region, normalized scatter determines the ratio of dark-field and attenuation signal contrast between these regions:

$$\begin{array}{c} \text{Norm}.\;\text{scatt}=\frac{{\left\langle \ln D\right\rangle }_{a}-{\left\langle \ln D\right\rangle }_{b}}{{\left\langle \ln T\right\rangle }_{a}-{\left\langle \ln T\right\rangle }_{b}} \end{array}$$ (22)

Dividing this value by the ratio of dark-field and attenuation noise levels then yields the ratio of dark-field and attenuation contrast-to-noise ratios between the two regions:

$$\begin{array}{c} \frac{\text{Norm}.\;\text{scatt}}{\sigma (\ln D)/\sigma (\ln T)}=\frac{\left[{\left\langle \ln D\right\rangle }_{a}-{\left\langle \ln D\right\rangle }_{b}\right]/\sigma \left(\ln D\right)}{\left[{\left\langle \ln T\right\rangle }_{a}-{\left\langle \ln T\right\rangle }_{b}\right]/\sigma \left(\ln T\right)}=\frac{{\text{CNR}}_{a,b}\left(\ln D\right)}{{\text{CNR}}_{a,b}\left(\ln T\right)}. \end{array}$$ (23)

Here, the simplification is used that the noise levels are approximately the same in both regions, which is appropriate if their signal levels deviate only slightly. Other than that, the result is independent of the exact difference in quantity of lung tissue between the regions. This allows comparing the abilities of both modalities to detect small differences in lung tissue.

With experimental values for *σ*(ln *D*)/*σ*(ln *T*) ranging between 5.1 and 7.8, and normalized scatter values in the vicinity of 0.42 to 0.56, it follows that ratios of dark-field and attenuation CNR for *in vivo* lung tissue are surprisingly low with approximately 5.4 to 11%, depending of ventilation level and the position in the thorax (cf. Table 3).

#### Influence of tissue overlap

An effect that was ignored in the preceding calculation is that the lung is superimposed by multiple organs in thorax radiography.

This occlusion leads to significant additional contrast not of diagnostic relevance in the conventional image. Very small tissue structures may also appear as “anatomical noise” in the conventional image. A distinction between diagnostically relevant and irrelevant features is then only possible using morphological information, e.g. by a trained radiologist. These effects are however much less relevant for the dark-field modality, as the lung is by far the most prominent source of dark-field signal in the thorax, and other structures are thus rendered nearly invisible. Unfortunately, a quantitative analysis of this effect is difficult due to a high variability of anatomical features between animals, and a strong dependence on the lung region under investigation.

However, we calculated the fraction *f* of total attenuation signal due to the lung according to Eq (16), which provides an indication for the relative impact of the lung in conventional radiography of the porcine thorax. For a given value of *f*, superimposed contrasts from other tissues may exceed the contrast of interest by a factor of (1 − *f*)/*f*. Maps and histograms of *f* are shown in S2 Fig: For the majority of the lung-covered area, less than *f* = 25% of the attenuation signal are due to the lung itself.

Note that the pneumothoraxes analyzed in [32], where CNR values between pneumothorax and adjacent lung were found to be significantly higher in the dark-field modality, were located in distal areas where *f* is especially low.

Although there are multiple differences for clinical thorax imaging (higher acceleration voltages and higher fractions of detected Compton-scattered intensities, as well as anatomical differences between porcine and human thoraxes), we believe that similarly low values for *f* would be achieved.

## Discussion

### Previous research

Knowing the dependence of dark-field signal on breathing cycle phase is important for translating the technique to clinical use. This dependence has not yet been systematically examined, however. For small-animal dark-field measurements, breathing was not paused during acquisition, and signal strength was thus effectively averaged over all phases of the breathing cycle. Fringe-scanning dark-field radiographs of pigs and humans, on the other hand, have been acquired with paused ventilation, since thorax motion during fringe-scanning acquisition leads to significant imaging artifacts. This resembles the clinical practice of thorax radiography, where patients are asked to hold their breath during image acquisition to minimize blurring.

Furthermore, observed variations in signal strength can be caused by both variations in projected lung thickness, as well as lung density. Radiographic information alone is unable to distinguish between these effects.

The effect of microstructural size variations in lung- or foam-like materials on dark-field signal has been examined in wave-optical simulations [37, 38], but an experimental verification with a human or large-animal thorax has, to our knowledge, not yet been presented.

In a recent study [32], lateral pneumothorax in pigs was found to appear in dark-field radiography with a higher CNR than in conventional X-ray. In conventional radiography, the contrast due to features of interest competes with contrasts from other superimposed structures, whereas this effect is largely absent in dark-field radiography. The relative importance of this advantage, compared to “pure” CNR performance was however not examined in that study.

### Methodology

In this work, we acquired *in vivo* thorax dark-field radiographs of two pigs. Three different stages in the breathing cycle were simulated by (paused) mechanical ventilation. Position and ventilation pressures were replicated for CT thorax imaging of the same animals. Segmentation, masking, and forward-projection of CT data yielded maps of lung thickness and water-equivalent height of the lung, in registration with the dark-field radiographs.

### Results from linear regression

Pixel-for-pixel correlation of logarithmic dark-field signal with both types of thickness maps yielded approximately linear relationships, motivating regression of linear functions, albeit with a non-zero axis intercept. We show that the nonzero intercepts from regression analysis can be explained by a deviation in the fraction of Compton-scattered radiation observed in the sample scans and the calibration scans for correcting visibility-hardening.

Dark-field signal increase per lung thickness retrieved from regression analysis is equivalent to the dark-field extinction coefficient as defined by Lynch et al. in [8], averaged over the entire lung. Regression results show that this coefficient is 42% (animal 1) / 33% (animal 2) lower at the highest ventilation pressure (20 mbar) than at the lowest one (2 mbar). Such a behavior is also expected for the logarithmic transmittance of the lung, as the (constant) amount of lung material is distributed over a greater projected area during inspiration. As the linear attenuation coefficient is proportional to mass density, it means that its average value over the entire lung must be inversely proportional to lung volume, which we could verify from CT measurements. The logarithmic dark-field signal however was found to decrease slightly more slowly with increasing lung volume.

We believe that this higher-than-expected dark-field activity may be due to additional alveolar recruitment, or changes of microstructural length scales at higher pressures: As the dark-field signal depends on the sample’s autocorrelation function of electron density, thinning alveolar walls or increases in alveolar diameter may affect the dark-field extinction coefficient. Both alveolar recruitment as well as uniform expansion (which is most clearly associated with microstructural length scale changes) are mechanisms involved in alveolar dynamics, and have e.g. been observed with optical coherence tomography [34, 35].

The second result from linear regression, dark-field signal increase per attenuation-equivalent water height *d*^H2O^, is proportional to the ratio of mean dark-field extinction coefficient and linear attenuation coefficient, i.e. an average “normalized scatter” coefficient of the lung. We find a slight increase of this ratio for rising ventilation pressure, which is consistent with the preceding results (slower decrease of dark-field extinction coefficient than linear attenuation coefficient for increasing pressure / lung volume).

In a recent publication [38], linear diffusion coefficients of human and murine lung tissue were calculated via wave-optical simulations of a simple lung microstructure model. Using our setup parameters, we converted their results to dark-field extinction coefficients, and find values of 1.94, 1.34, and 1.04 m^-1^ (for 200, 300, and 400 μm alveolar diameter), which are smaller than the values presented here by factors between 1.2 and 4.4.

However, these simulations were performed for a 120 kV tube spectrum. Although a precise conversion to a different flux and visibility spectra would require a new simulation, we can make a simple approximation: Mean energy for the simulations (*E*_mean_ = 64.5 keV) exceed the one used here (42.1 keV) by a factor of 1.53. Given the proportionality of −ln *D* with *E*^−2^, and ignoring any deviations in both visibility spectrum and autocorrelation length, we expect our signal values to be greater by a factor of 2.35, which is in good agreement with the factors mentioned above.

### Results from CNR analysis

The internal structure of the healthy lung causes it to generate a strong dark-field signal and a weak attenuation signal, considering the organ’s overall size. It is thus plausible to suspect that the dark-field contrast due to a given amount of lung is so much greater than the attenuation contrast that it is able to compensate the intrinsically greater noise levels in the dark-field modality, and thereby explain the findings from [32] of greater dark-field CNR for pneumothorax than attenuation CNR.

The ratio of dark-field and attenuation CNR values can be calculated from the “normalized scatter” coefficient and the ratio of noise levels in both modalities, cf. Eq (23). Noise levels were however not directly measured by analysis of the pig thorax scans. To exclude superposition effects, we constructed a phantom from material slabs of uniform thickness, which generated dark-field and attenuation signal levels comparable to a previously imaged porcine thorax. Noise levels were then measured by ROI analysis in both modalities. The ratio of dark-field and attenuation standard deviations was compared to theoretical calculations (propagation of shot noise to processed modalities).

The measured values for these ratios were found to exceed the theoretical results. We suspect that this is partly due to the existence of “structural noise” in the dark-field images, even for the uniform chloroprene sheets used in the phantom. As dark-field signal is intrinsically caused by a spatial variation of electron density, we think that structural noise is an inevitable side-effect if this variation extends to the length scale of effective pixel size (as may also be the case for lung tissue).

Contrary to expectations, the ratios of dark-field CNR and attenuation CNR for a given height difference of lung tissue were found to be far below 1 (5.4 to 11%), which stands in contrast to the promising results from [32]. In chest X-ray however, the lung is superimposed by attenuation contrasts from other, more strongly attenuating materials, which is not simulated by the presented phantom. As the impact of this effect on CNR depends on anatomical structure and is thus highly variable, a precise analysis is difficult. We estimate the magnitude of this effect by calculating the fraction of attenuation signal due to the lung in one of the pig thorax images. The lung is found to contribute less than 25% to total attenuation in the majority of the lung area (whereas it is contributes nearly the entire dark-field signal). It is thus clear that the potential impact of tissue superposition is very high.

## Conclusion and outlook

In this work, we have combined imaging data from dark-field chest radiography and thorax CT of two living pigs at three different ventilation pressures. Furthermore, we acquired dark-field radiographs of a simple phantom and performed theoretical calculations to evaluate noise levels in the dark-field and attenuation modalities. The main findings are as follows:

- Correlation of dark-field signal with the lung thickness retrieved from CT showed an approximately **linear dependence**. It allowed calculation of the **lungs’ mean dark-field extinction coefficient** and revealed its **strong dependence on lung ventilation** (variation by up to 42%). The numerical values are in good agreement with wave-optical simulations of a simple lung tissue model [38].
- Correlation of dark-field signal with attenuation-equivalent water height retrieved from CT was also approximately **linear**. Combined with simulated values for the effective linear attenuation coefficient of water, this allowed calculation of the **mean “normalized scatter” value of lung tissue**, which we found to be **nearly independent of ventilation pressure**.
- Measurement of phantom data and theoretical calculations revealed that dark-field noise levels are **five to eight times higher** than those in conventional radiography.
- By combining “normalized scatter” values with the noise levels ratios, we found that **dark-field CNR for a given height difference of lung tissue is only 5 to 10% of the attenuation CNR**. Given contradictory findings in a previous study [32], we conclude that the **influence of anatomical noise is much higher in conventional radiography** and may significantly improve the relative CNR performance of dark-field radiography.

These findings have a high relevance for the clinical interpretation of dark-field thorax radiographs: The phase in the breathing cycle when the image is acquired may affect its diagnostic benefit due to the variation of signal strength and projected lung size. However, the presented results are also subject to some limitations:

- **Microstructural parameters may differ between human and porcine lungs**. However, the mechanical processes of breathing are very similar. Accordingly, we expect quantitative values to deviate for human lungs measured *in vivo*, but we predict relative trends over the breathing cycle to be comparable. Furthermore, we think the dark-field extinction coefficient’s approximate proportionality with inverse lung volume will also apply for human lungs, since the microstructural processes during breathing are likely very similar in human and porcine lungs.
- **Transferability of these results to other setups and imaging parameters may be limited**.
  - The determination of electron density autocorrelation functions of lung tissue at various ventilation pressures would allow prediction of signal levels for arbitrary X-ray energies and phase-sensitivities [7, 8]. However, this is not currently feasible as there is no universally accepted model of alveolar micromechanics in the living lung [34].
  - For the present setup and imaging parameters, sampled autocorrelation lengths (named *pd* in [7], *d* in [8]) are in the vicinity of 0.6 μm. It is important to consider that the range of electron density autocorrelation lengths suitable for human thorax imaging is **limited by SNR considerations**: Much higher values than e.g. 1 μm would achieve very low visibility (and thus, high noise levels) behind a human lung, whereas much lower values yield only a very weak dark-field signal.
  - Since we have not observed abrupt changes in dark-field signal while varying ventilation pressure (and thus, varying dimensions in alveolar microstructure), we think that the autocorrelation function of electron density is smooth in the vicinity of the sampled correlation length values. Furthermore, a large amount of random variation is probably smoothed out due to averaging along the length of the X-ray path through the lung. Thus, we expect setups with similar ranges of sampled autocorrelation lengths to achieve results comparable to the ones presented here.
- In the presented measurements, lung volume at 20 mbar was 60 to 80% higher than at 2 mbar. In a clinical setting with a freely breathing patient however, **much greater relative changes in volume could be achieved**. It is yet to be shown whether the examined correlations still apply for these more extreme levels of inhalation. However, this information could probably only be retrieved from a freely-breathing patient, e.g. in a clinical study of dark-field radiography.

## Supporting information

S1 Fig Bivariate histograms of dark-field signal vs. lung thickness and attenuation signal. Dependence of dark-field signal on *d*^lung^ and *d*^H2O^, for all measurements in the study, including those shown in Fig 6. Rows 1, 2: Data from Animal 1; Rows 3, 4: Data from Animal 2. Rows 1, 3: Plots of −ln *D* vs. *d*^lung^; Rows 2, 4: Plots of −ln *D* vs. *d*^H2O^. Left / middle / right column: Ventilation pressures of 2 / 11 / 20 mbar.
(TIF)

S2 Fig Fraction *f* of the attenuation signal due to the lung in the porcine thorax at 60 kV (animal 1). Ratios of lung attenuation signal and total attenuation signal are shown as spatial maps and histograms for two different values of airway pressure. Calculation via Eq (16) in the main text. (A), (C) Exhalation (2 mbar). (B), (D) Inhalation (20 mbar). In most image areas, the attenuation signal is dominated by organs other than the lung.
(TIF)

S3 Fig Elastic registration of forward-projected CT data with dark-field radiographs. Projected lung thickness *d*^lung^ (animal 1, 2 mbar), before (blue) and after (red) elastic registration to fringe-scanning data. Arrows indicate the magnitude and direction of local shifts. Minimum and maximum shifts in the area of the lung are 3.2 and 27.0 pixels. Using the effective pixel size of the dark-field setup in a plane 10 cm above the sample table, these correspond to distances of 1.1 and 9.5 mm. Mean displacement in the lung area is 17.3 pixels (6.1 mm).
(TIF)

S1 File Checklist for ARRIVE (Animal Research: Reporting of *In Vivo* Experiments) guidelines. Guidelines designed by NC3Rs (National Centre for the Replacement, Refinement & Reduction of Animals in Research) to improve reporting of research using animals, see https://www.nc3rs.org.uk/arrive-guidelines.
(PDF)
