# Supplementary figures and images for: Contrast-to-noise ratios and thickness-normalized, ventilation-dependent signal levels in dark-field and conventional in vivo thorax radiographs of two pigs
Source: PLoS One. 2019 Jun 3;14(6):e0217858. doi: 10.1371/journal.pone.0217858 (PMC6546243; doi:10.1371/journal.pone.0217858)

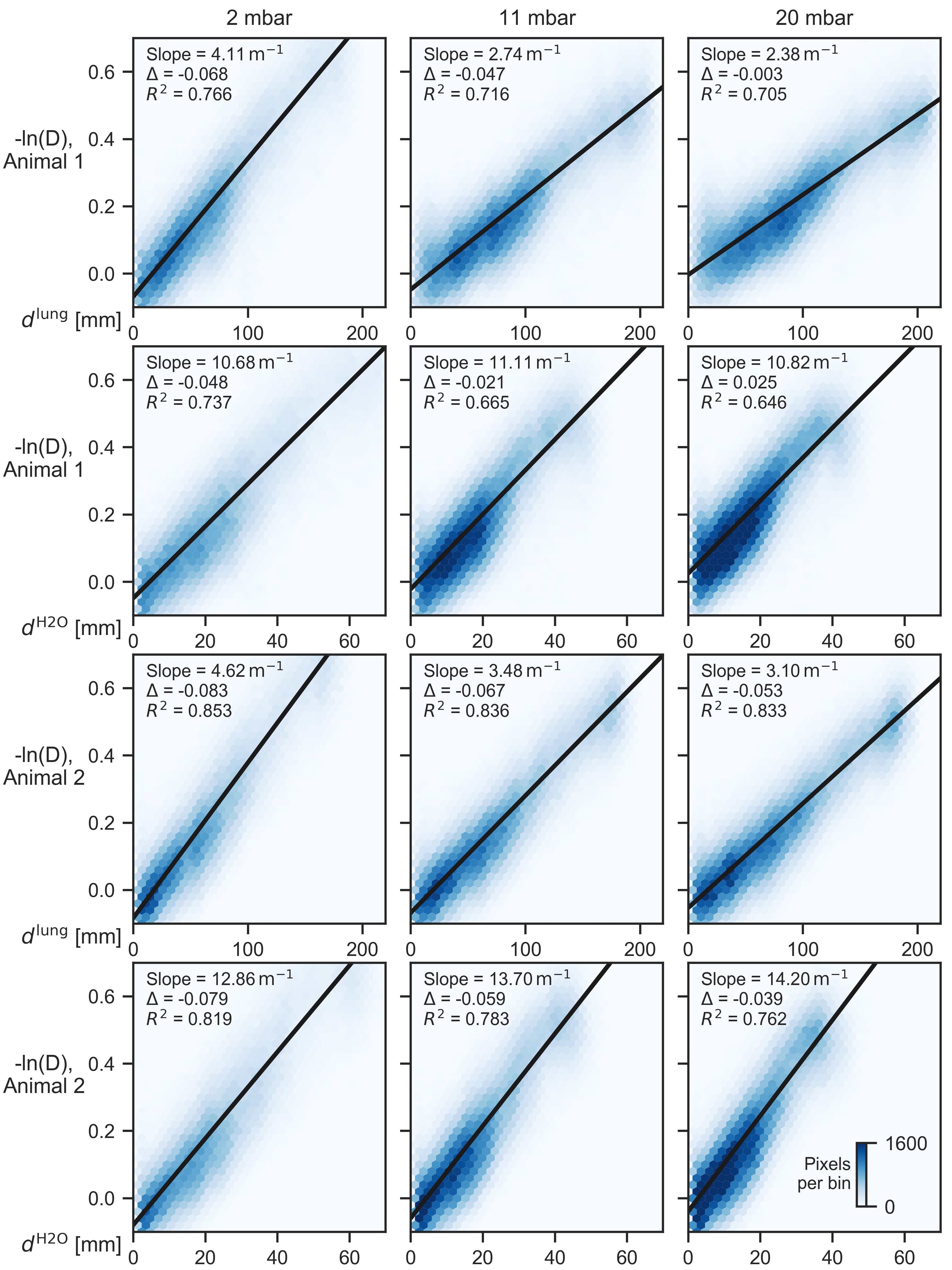

Supplement: S1 Fig — Dependence of dark-field signal on dlung and dH2O, for all measurements in the study, including those shown in Fig 6. Rows 1, 2: Data from Animal 1; Rows 3, 4: Data from Animal 2. Rows 1, 3: Plots of −ln D vs. dlung; Rows 2, 4: Plots of −ln D vs. dH2O. Left / middle / right column: Ventilation pressures of 2 / 11 / 20 mbar. (TIF) [file pone.0217858.s001.tif]

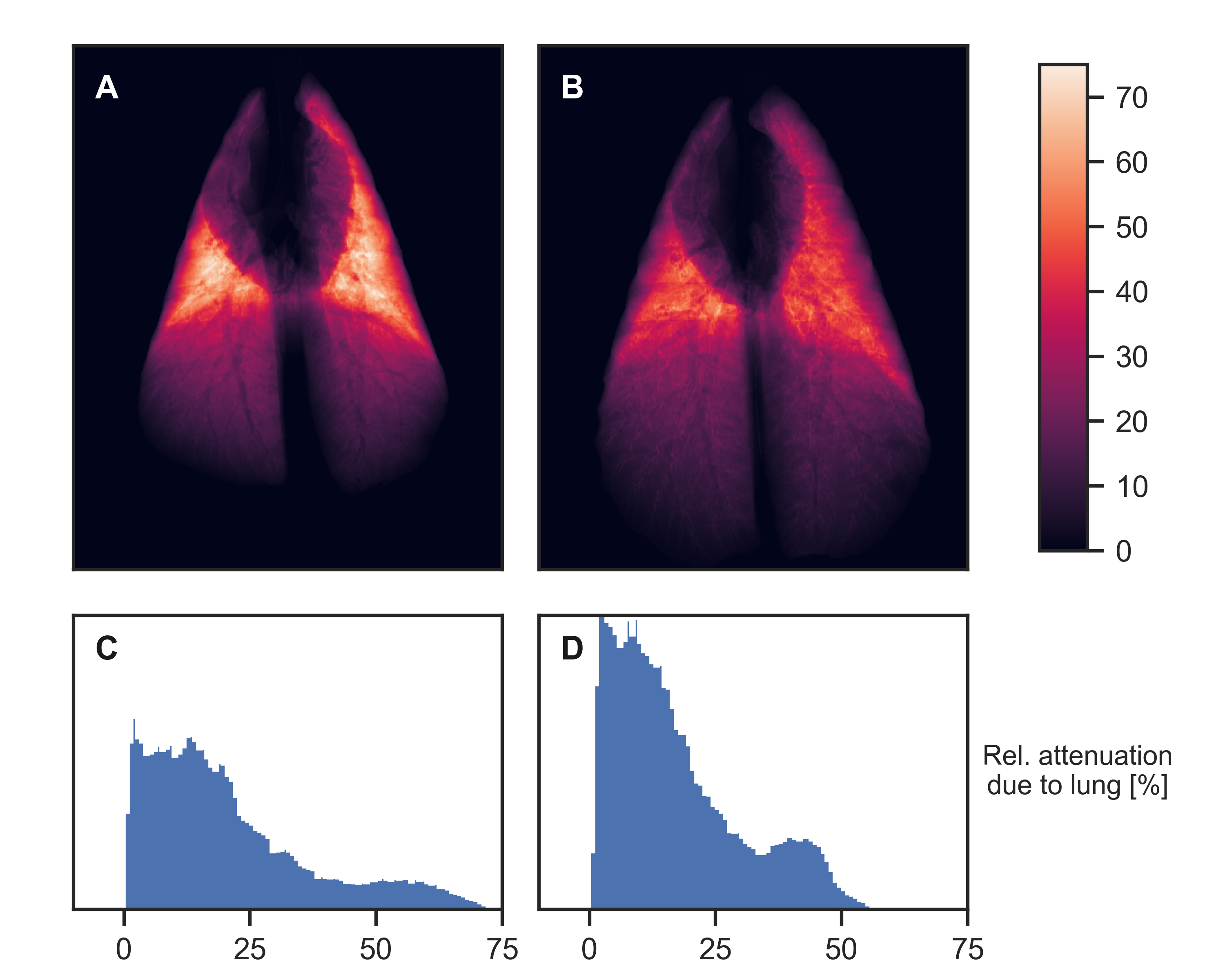

Supplement: S2 Fig — Ratios of lung attenuation signal and total attenuation signal are shown as spatial maps and histograms for two different values of airway pressure. Calculation via Eq (16) in the main text. (A), (C) Exhalation (2 mbar). (B), (D) Inhalation (20 mbar). In most image areas, the attenuation signal is dominated by organs other than the lung. (TIF) [file pone.0217858.s002.tif]

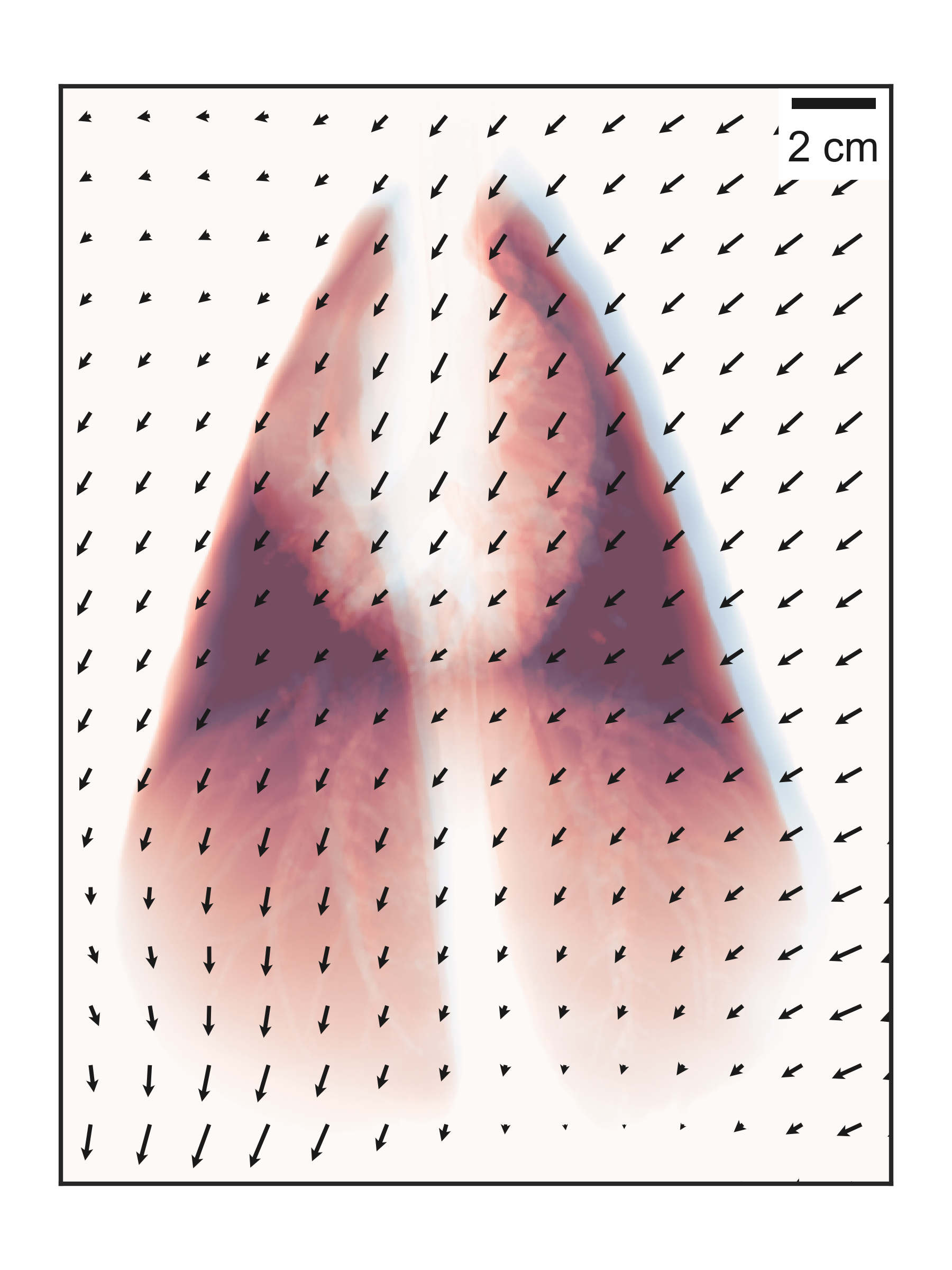

Supplement: S3 Fig — Projected lung thickness dlung (animal 1, 2 mbar), before (blue) and after (red) elastic registration to fringe-scanning data. Arrows indicate the magnitude and direction of local shifts. Minimum and maximum shifts in the area of the lung are 3.2 and 27.0 pixels. Using the effective pixel size of the dark-field setup in a plane 10 cm above the sample table, these correspond to distances of 1.1 and 9.5 mm. Mean displacement in the lung area is 17.3 pixels (6.1 mm). (TIF) [file pone.0217858.s003.tif]
